# Inducing amnesia through systemic suppression

**DOI:** 10.1038/ncomms11003

**Published:** 2016-03-15

**Authors:** Justin C. Hulbert, Richard N. Henson, Michael C. Anderson

**Affiliations:** 1 Bard College, Psychology Program, PO Box 5000, Annandale-on-Hudson, New York 12504, USA; 2 Medical Research Council, Cognition and Brain Sciences Unit, 15 Chaucer Road, Cambridge CB2 7EF, UK; 3 Behavioural and Clinical Neurosciences Institute, Sir William Hardy Building, Downing Street, University of Cambridge, Cambridge CB2 3EB, UK

## Abstract

Hippocampal damage profoundly disrupts the ability to store new memories of life events. Amnesic windows might also occur in healthy people due to disturbed hippocampal function arising during mental processes that systemically reduce hippocampal activity. Intentionally suppressing memory retrieval (retrieval stopping) reduces hippocampal activity via control mechanisms mediated by the lateral prefrontal cortex. Here we show that when people suppress retrieval given a reminder of an unwanted memory, they are considerably more likely to forget unrelated experiences from periods surrounding suppression. This amnesic shadow follows a dose-response function, becomes more pronounced after practice suppressing retrieval, exhibits characteristics indicating disturbed hippocampal function, and is predicted by reduced hippocampal activity. These findings indicate that stopping retrieval engages a suppression mechanism that broadly compromises hippocampal processes and that hippocampal stabilization processes can be interrupted strategically. Cognitively triggered amnesia constitutes an unrecognized forgetting process that may account for otherwise unexplained memory lapses following trauma.

## Abstract (web summary)

Suppressing an unwanted memory reduces hippocampal activity and makes people more likely to forget the experience. Here, the authors show that suppressing past events induces an "amnesic shadow" for experiences near in time to suppression, consistent with a global disruption to hippocampal function.

## Graphical abstract

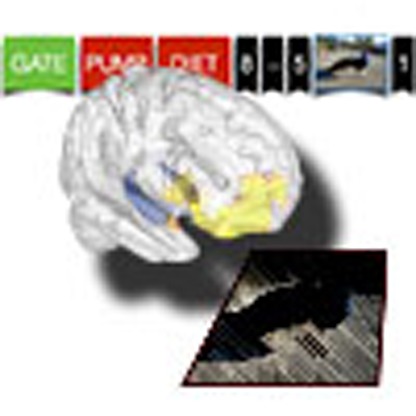

Trying to forget the past may inadvertently cause amnesia for the present. This surprising possibility follows from the neural mechanisms underlying memory formation and motivated forgetting. Decades of research on memory formation show that the hippocampus is essential for constructing new episodic memories. Hippocampal damage irreversibly harms people's ability to store new memories, causing profound amnesia for life's events^1^,^2^. Reversibly disturbing the hippocampus through optogenetic, electrical and pharmacological interventions temporarily disrupts memory formation^3^,^4^. Research on motivated forgetting, on the other hand, indicates that people often downregulate hippocampal activity through cognitive control when they are reminded of an unwelcome event and try to stop retrieval^5^,^6^,^7^,^8^,^9^,^10^,^11^. Together, these findings imply a striking possibility: if stopping (suppressing) episodic retrieval reduces hippocampal activity, this may broadly disturb all hippocampal functions, including—critically—processes necessary to form and retain new, stable memories. Retrieval suppression may, in essence, induce a transient ‘virtual lesion', leaving in its wake, an amnesic shadow for any experiences—whether related or not to the memory being suppressed—that simply have the misfortune of happening near in time to efforts to forget.

To test this hypothesis, we measured how well people remembered experiences near in time to their efforts to retrieve or suppress an unrelated memory. To induce retrieval and suppression, we adapted the Think/No-Think (TNT) procedure used to study how people control unwanted memories, yielding a new hippocampal modulation paradigm (HM Paradigm, Fig. 1). In the TNT procedure, people perform trials requiring them to attend to a reminder of a past event; for each reminder, they are cued to retrieve the associated memory (Think trials), or to suppress its retrieval (No-Think trials). Suppressing retrieval in response to a strong reminder reduces blood–oxygen-level dependent activation in the hippocampus and impairs retention of the suppressed memory^5^,^6^,^7^,^8^,^9^,^10^,^11^. These reductions originate from inhibitory control processes supported by the dorsolateral prefrontal cortex^6^,^7^,^8^,^9^,^10^,^11^. Retrieving memories, in contrast, increases hippocampal activity^12^ and often facilitates later recall^13^. Thus, hippocampal activity can be modulated according to task goals, though practice at suppressing retrieval is often necessary to achieve hippocampal reductions^6^. A key assumption of the present work is that this modulation does not target particular memories, which may be a person's goal, but rather reflects a broadly targeted suppression (hereinafter, ‘systemic suppression') of regional activity in the hippocampus that generally disrupts other memory functions supported by this region. For instance, beyond simply disrupting episodic retrieval, systemically suppressing hippocampal activity may also prevent freshly encoded stimulus input from generating new hippocampal traces (that is, hippocampal encoding) or, instead may disrupt existing hippocampal encodings, preempting their transformation into stable episodic memories (that is, stabilization processes).

To examine how suppression affected memories near in time, we inserted novel ‘innocent bystander' stimuli between Think or No-Think trials (Fig. 1). Unbeknownst to participants, we planned to test their memory for these items after the hippocampal modulation task. We selected bystanders that were unrelated to the memories people controlled during Think and No-Think trials, allowing us to assess whether suppression broadly affected memories near in time. For each bystander, participants made a simple semantic encoding decision. Importantly, both before and after each bystander, we inserted ‘buffer' intervals during which participants viewed a series of digits and decided whether each was odd or even. The buffer intervals inserted before bystander stimuli ensured that the same task always preceded bystanders, holding the distraction caused by switching between tasks constant across conditions (see Methods section and Supplementary Information for further discussion); buffer intervals following bystanders functioned to interrupt participants' lingering thoughts about the bystander, ensuring that voluntary encoding processes ceased before the next suppression or retrieval trial^14^. Because the buffer trials before and after bystanders were present uniformly in all conditions, any observed differences in later bystander recall across conditions must arise from neighbouring suppression and retrieval trials and not from the immediately surrounding buffer task.

Our amnesic shadow hypothesis predicts memory lapses for experiences occurring near in time to retrieval suppression, when suppression has impaired hippocampus-dependent functioning. Suppression should therefore reduce episodic memory indices for bystanders, such as contextual binding^15^ and recollection, which depend on the hippocampus and are impaired in amnesia^16^. The worst forgetting should arise when suppression abuts an event on both sides (two suppression doses); an intermediate amount when it abuts one side (one dose); and the best retention when it abuts neither side (zero dose). We focused our initial analyses on bystanders in the second half of the hippocampal modulation session, after participants had practised suppressing—the same period that suppression-related hippocampal activity has been driven below pretrial baseline in prior work^6^. We found that suppressing episodic retrieval of an unwanted memory induces an amnesic shadow for experiences occurring near in time to efforts to suppress, and that this forgetting is related to the controlled reduction of hippocampal activity.

## Results

### A lasting reduction in episodic memory

**Figure 1:**
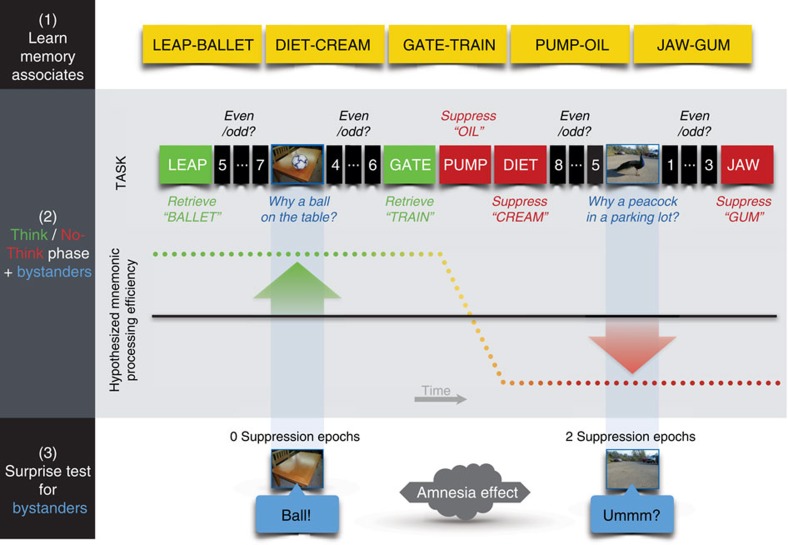
Hippocampal modulation (HM) paradigm and predictions. After studying word pairs (phase 1), participants perform trials requiring that they either retrieve (green) or suppress (red) retrieval of the second word in each pair, given the first word. Inserted between these trials are novel ‘bystander' pictures that participants encode by silently answering the question, ‘Why is the pictured object in this location?' Even/odd buffer judgments performed before and after bystanders match the immediate task context across retrieve and suppress trials. The line graph illustrates the predicted efficacy of hippocampus-dependent memory processes (‘mnemonic processing efficiency') during the above events. Of interest is whether surrounding bystanders with suppression trials affects later memory for bystander pictures, causing an ‘amnesic shadow.' This is assessed in phase 3, in which the participant must recall the associated object for each context scene.

In experiment one, the to-be-controlled memories were word pairs and the bystanders were novel photographs, each presenting a central object in a background setting. The relation between the object and the setting was not predictable (for example, a peacock in a parking lot; Fig. 1). When each photograph appeared, participants imagined how the object came to be in the setting and rated the difficulty of constructing the explanation—a task that encouraged episodic associations between the object and its setting. After the hippocampal modulation phase, we displayed each background and asked participants to remember what object had appeared in it.

**Figure 2:**
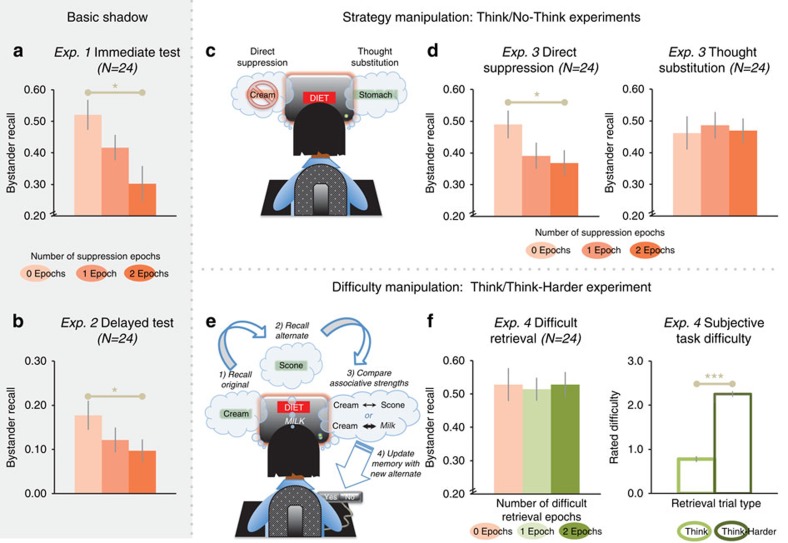
Amnesic shadow in cued recall. (**a**) Immediate cued-recall accuracy for bystanders by number of adjacent suppression epochs. Difference between the left- (peach colour) and right-most (dark orange) bars reveals an amnesic shadow (*F*-test). (**b**) Shadow observed after 24 h delay. (**c**) Experiment 3's two No-Think strategies. (**d**) Direct suppression, not thought substitution, caused a shadow. (**e**) Experiment 4 replaced No-Think trials with a difficult ‘Think-Harder' task. (**f**) No shadow was observed in experiment 4 (left subpanel), despite a significant difficulty disparity across types of surrounding retrieval epochs (right subpanel, paired *t*-test). Error bars reflect within-participant s.e.m. *P<0.05; ***P<0.001.

Participants' memory suffered for bystander scenes presented between suppression trials (Fig. 2a). Relative to bystanders dosed with zero suppressions, object recall (for example, peacock) was worse for scenes dosed with two epochs (F_1,20_=7.63, *P*=0.012). Remarkably, object recall showed a 42% proportional reduction, relative to the zero-dose condition. Experiment 2 replicated this pattern (Fig. 2b; F_1,20_=4.65, *P*=0.043) with a 24-h delay between suppression and test, revealing a similar 45% proportional recall reduction (see Supplementary Fig. 1 and Supplementary Table 1 for an overview of this and later experiments).

It was necessary to consider whether memory lapses might simply reflect differences in processing time on bystander items, perhaps owing to differences in distraction following suppression and retrieval trials. No evidence of differential processing was found, however. On the semantic decision task (Supplementary Fig. 2a), participants spent comparable time across the three conditions in making their ratings (F_2,38_=0.28, *P*=0.757), and the rating decisions themselves did not differ (F_2,38_=1.05, *P*=0.358). Moreover, the intervening buffer task (Supplementary Fig. 2b) should have been affected by such distraction, but performance on it differed neither in speed nor accuracy as a function of whether it was performed after a retrieval or suppression trial (trials occurring before bystander presentation: reaction time, F_1,20_=0.11, *P*=0.743; accuracy, F_1,20_=0.05, *P*=0.829; trials occurring afterwards: reaction time, F_1,20_=2.18, *P*=0.156; accuracy, F_1,20_=1.69, *P*=0.209). This conclusion held even after aggregating reaction times from the hundreds of participants across all studies in this article (representing 14,000–16,000 trials per condition; see Supplementary Table 2 and Supplementary Data for detailed analyses). The buffer task data thus provide little support for the possibility that participants were differentially distracted by the preceding suppression or retrieval tasks during bystander presentation. The large memory reduction observed in experiment 1 is thus consistent with the hypothesized dose-dependent disruption of hippocampus-dependent memory processes caused by the surrounding suppression periods.

### Specificity of deficits to suppression

According to our hypothesis, the amnesic shadow is tied to control processes that systemically reduce hippocampal activity. Recent work indicates that people can control retrieval of an unwanted memory in two ways. On one hand, they can stop retrieval entirely (that is, ‘direct suppression'), which reduces hippocampal activity; alternatively, people can avoid retrieving a memory by recalling a ‘substitute thought,' which does not reduce hippocampal activity^8^. If so, bystanders inserted between direct suppression trials should exhibit a shadow, but those inserted between thought substitution trials should not. Experiment 3 confirmed this prediction (0 versus 2 doses for the direct suppression group, F_1,20_=4.91, *P*=0.038, 25% proportional reduction; for thought substitution, F_1,20_=0.01, *P*=0.916, 2% proportional facilitation; Fig. 2c,d). Thus, despite having the same intention to avoid retrieving No-Think items, the substitution group showed no shadow.

Although retrieval suppression may trigger memory lapses, perhaps any suitably difficult task would also make people forgetful. Perhaps suppression simply distracts participants, despite our including buffer tasks intended to absorb the effects of such distraction. If so, replacing retrieval suppression with an exceptionally difficult retrieval task also should induce memory lapses, even when such a task should not reduce hippocampal activity. We tested this idea in experiment 4, in which we replaced No-Think trials with an exceptionally difficult ‘Think-Harder' task. On ‘Think-Harder' trials, participants had to retrieve two studied associates for each cue while also viewing a new word; moreover, they needed to assess the new word's relatedness to the retrieved associates, and, under certain conditions, replace an associate with the new word on future trials—in other words, a task requiring multiple retrievals, new learning and memory updating (Fig. 2e). Despite a wide disparity in perceived difficulty across Think and Think-Harder trials (Fig. 2f, right), retention of bystander events did not vary according to whether they were surrounded by easy or hard trials (Fig. 2f, left; F_1,32_<0.01, *P*>0.999), indicating that suppression-specific processes cause forgetting, not task difficulty.

**Figure 3:**
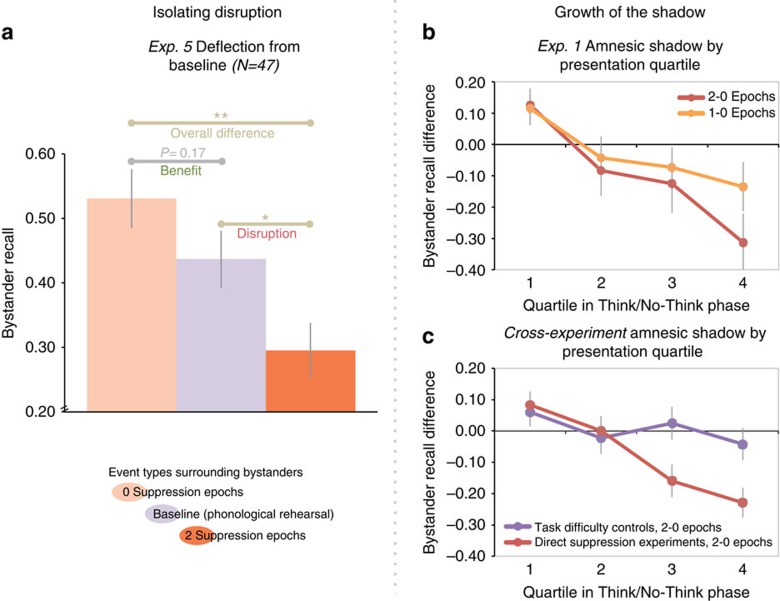
Nature and growth of the amnesic shadow. (**a**) Cued-recall accuracy in experiment 5 for the zero- and two-dose conditions compared with baseline (purple) with *F*-tests. Whereas the above-baseline benefit was not significant, below-baseline disruption was. (**b**) The amnesic shadow (negative deflection of the red line away from the *x* axis) grew across quartiles, consistent with a practice effect. (**c**) Unlike the growing shadow apparent across immediate cued-recall studies (red line), thought substitution (experiment 3), Think-Harder (experiment 4) and baseline (experiment 5) studies showed no such growth with practice (purple line). Error bars reflect s.e.m. *P<0.05; **P<0.01.

Although consistent with an amnesic shadow, our findings could also reflect improved memory for bystanders in the zero-dose condition caused by nearby retrievals^17^. To test this, experiment 5 compared memory for bystanders surrounded by suppressions or retrievals with memory in a baseline group. In this group, we replaced Think and No-Think cues with trial-unique nonsense words that participants rehearsed in a forward (green trials) or backward (red trials) direction—tasks selected because they do not engage episodic retrieval. Otherwise, we matched the hippocampal modulation and baseline procedures, including word-pair training, red and green prompts, timing and trial structure (Methods section, Supplementary Methods). The baseline task thus measured bystander memory uncontaminated by processes that modulate hippocampal activity. Because surrounding bystanders by forward or backward rehearsal did not affect recall (F<1), we averaged across these conditions, yielding one baseline. Compared with this baseline, surrounding bystanders with retrievals tended to improve recall, though not reliably (F_1,39_=2.00, *P*=0.166); surrounding them with suppressions, however, reliably harmed memory (Fig. 3a; F_1,39_=5.29, *P*=0.027), yielding a robust overall shadow (zero- versus two-dose conditions, F_1,20_=13.71, *P*=0.001, a 44% proportional reduction). These findings indicate that the shadow owes, in significant part, to impairment prompted by suppression.

### Growth with repeated suppression

If the amnesic shadow tracks hippocampal modulation, memory lapses may increase with repeated suppression^11^. Evidence indicates that hippocampal downregulation grows with practice at suppressing retrieval^6^. This growth may be part of a qualitative shift in the fronto-posterior networks supporting control, perhaps reflecting the tuning of suppression in response to memory intrusions^6^,^7^. Consistent with this, forgetting grew across quartiles of the hippocampal modulation task in experiment 1 (Fig. 3b). Indeed, across all immediate recall studies involving direct suppression, practice increased amnesia: The first half of the task showed no shadow (F_1,60_=1.87, *P*=0.177), whereas the second half did (F_1,60_=24.90, *P*<0.001; interaction across halves, F_1,60_=26.23, *P*<0.001), and amnesia linearly increased across quartiles (Fig. 3c, F_1,60_=29.27, *P*<0.001). This linear decline largely remained after 24 h (experiment 2, F_1,20_=3.65, *P*=0.071). In contrast, in our control experiments (thought substitution, Think/Think-Harder and phonological baseline conditions), amnesia showed no such linear development (Fig. 3c; F_1,71_=1.38, *P*=0.244), and the build-up was greater for direct suppression than for control studies (interaction of linear trends, F_1,147_=7.99, *P*=0.005).

### Selective deficits for episodic context

**Figure 4:**
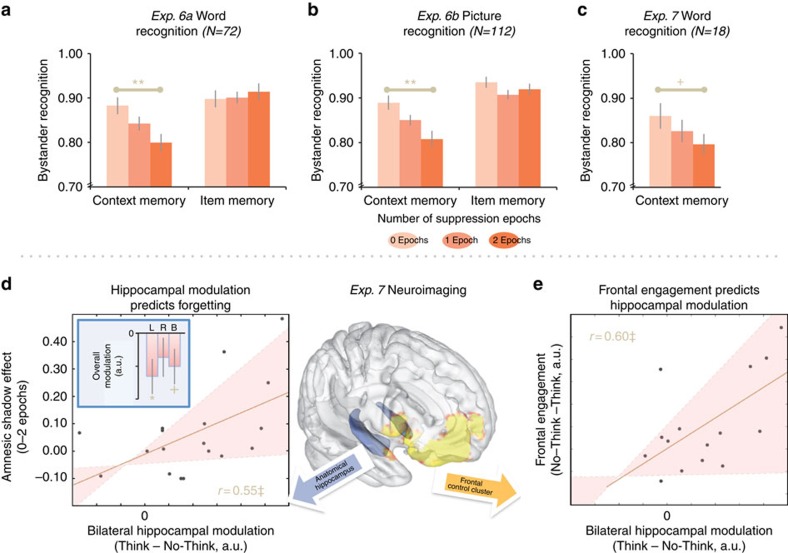
Amnesic shadow in recognition. (**a**) Memory for context, not items, is impaired for words (*F*-test). (**b**) Replication with pictures. (**c**) Context amnesia in experiment 7. (**d**) Right-most inset bar illustrates mean (error bar, s.e.m.) bilateral hippocampal modulation (blue anatomical region of interest seen in adjoining glass brain) during suppression, relative to retrieval in arbitrary units (a.u.). Modulation (two-tailed *t*-tests) in left and right hippocampi also is plotted separately for exploratory purposes (with Bonferroni correction for multiple corrections across the two hemispheres). Across participants, modulation predicted context amnesia (outlier-skipped Pearson bootstrap 95% confidence interval (CI): (0.13, 0.79)). Q (**e**) Suppression-related frontal engagement (yellow No-Think>Think functional mask) predicted hippocampal modulation (bootstrap CI: (0.03, 0.87)). Robust correlation removed bivariate outliers from relevant analysis/plot. Error bars for behaviour (**a**–**c**) reflect within-participant s.e.m.; red bands, 95% CI; ‡significant correlation by bootstrap test; *P<0.05; **P<0.01; +*P*≤0.10.

If hippocampal suppression triggers an amnesic shadow, recognizing bystander stimuli also should suffer, particularly when it depends on hippocampal representations. In many dual-process recognition models, the hippocampus helps form traces that enable people to consciously and vividly remember the particulars of an event^12^,^18^,^19^, whereas this structure may be relatively less involved in simply recognizing that the item had been encountered previously. People may therefore forget bystanders when recognition requires details of episodic context, more than basic item recognition. Experiment 6 evaluated this using a test that attempted to ascertain the relative contributions of context and item recognition^20^. We replaced bystander scenes with words or photographs of single items. Participants processed these items by judging whether each was animate or inanimate (animacy task), or instead whether it was pleasant or unpleasant (pleasantness task). On the recognition test, we measured two things for each bystander: people first rated whether they had viewed the item before (item recognition); they then decided which judgment they made about it (context memory; Supplementary Methods). Remarkably, whereas participants could recognize the items without difficulty (F_1,152_<0.01, *P*=0.984, no shadow), they exhibited a robust shadow in context memory (F_1,152_=18.58, *P*<0.001; interaction of shadow with measure, F_1.89,286.58_=10.82, *P*<0.001; Fig. 4a,b displays results separately for words and pictures). As in prior experiments, the context deficit grew linearly over quartiles (F_1,152_=6.65, *P*=0.011, Supplementary Fig. 3), unlike item memory (F_1,152_=0.143, *P*=0.706). The complete lack of a shadow for item memory is surprising, given that hippocampal lesions can impair item memory, though to a much lesser degree than context memory^21^,^22^. Nevertheless, the greater amnesic deficit in context memory is compatible with those dual-process models that ascribe the recognition of strong, context-rich memories disproportionally to the hippocampus^16^,^19^.

### Anterograde and retrograde components

Hippocampal amnesia patients experience profound memory loss^1^ for events occurring after their lesion (anterograde amnesia) and events occurring shortly before it (retrograde amnesia). To determine whether suppression induced parallel effects, we compared memory for bystanders that followed (anterograde) or preceded (retrograde) a single suppression dose to items not abutted by suppression (zero-dose condition). Across experiments, both effects occurred for immediate recall (anterograde, F_1,60_=23.68, *P*<0.001; retrograde, F_1,60_=8.06, *P*=0.006) and context recognition (retrograde, F_1,152_=10.08, *P*=0.002), though the anterograde effect did not reach significance on the latter measure (F_1,152_=2.10, *P*=0.150). Neither of these effects varied reliably across immediate and delayed tests (Fs<1), and indications of both effects occurred relative to baseline (Supplementary Data). Together, these components contribute to the dose-response effects observed across studies (0 versus 1 suppression dose in recall, F_1,60_=18.06, *P*<0.001; in recognition, F_1,152_=7.40, *P*=0.007; 1 versus 2 doses in recall, F_1,60_=4.59, *P*=0.036; in recognition, F_1,152_=6.29, *P*=0.013). The retrograde effect further highlights that memory lapses are unlikely due to inattention to bystanders, given that suppression's effect also occurred after bystander presentation. Rather, bystanders likely suffer from disrupted hippocampal function that interrupts stabilization^23^.

### Relation to hippocampal modulation

Experiment 7 confirmed the presumed relation of the amnesic shadow to suppression-induced hippocampal modulation using functional magnetic resonance imaging (fMRI). Adapting the recognition methods of experiment 6 (Methods section, Supplementary Methods), we observed a context recognition shadow under similar conditions in Experiment 7 (Fig. 4c, see Supplementary Methods and Supplementary Data for details). Prior work led us to expect this amnesic shadow would be related to hippocampal modulation driven by right lateral prefrontal cortex^6^,^8^,^9^,^10^,^11^,^24^. In line with this, retrieval suppression during No-Think trials engaged a cluster of right-lateralized cognitive control regions including the right middle and inferior frontal gyri (Fig. 4, yellow cluster in brain map) and also reduced overall hippocampal activity, primarily on the left (Fig. 4d inset: our *a priori* bilateral anatomical hippocampal region of interest (ROI), *t*(17)=−1.88, *P*=0.078). Right prefrontal activations also correlated with reductions in hippocampal activity (correlation with bilateral anatomical hippocampus, Pearson skipped *r*=0.60), echoing prior findings establishing right-lateralized top–down control of hippocampal activity^8^,^9^,^10^. Critically, hippocampal reductions during the TNT task positively predicted the extent of context amnesia for bystander stimuli (robust correlation with bilateral anatomical ROI, *r*=0.55), confirming the hypothesized link between hippocampal modulation and the amnesic shadow.

## Discussion

Together, our findings reveal that temporary suppression of hippocampal activity induced through cognitive control creates windows of amnesia in healthy people. Suppressing retrieval triggered enduring memory loss for unrelated ‘bystander' events, which showed up to a 45% proportional memory deficit. Strikingly, people forgot events they had fully attended to and processed deeply, and that were unrelated to the memories they suppressed. The amnesic shadow showed a dose-response function, occurred in both temporal directions, and increased with practice at stopping retrieval in both recall and context recognition—but not item recognition; and the shadow was not produced by distraction from other types of difficult tasks. Interestingly, controlling awareness of an unwanted memory through thought substitution rather than suppression produced no shadow. We predicted these characteristics from established properties of the hippocampus and confirmed a link to hippocampal modulation with fMRI.

The factors governing whether and how memories are disturbed need clarification. Whether memories are disturbed may depend on how close in time their encoding is to suppression episodes, perhaps showing a temporal gradient (though even older memories may suffer if reactivated recently)^25^. How memories are disturbed may depend on suppression-specific processes; alternatively, other tasks that reduce hippocampal activity may also disturb memory formation. Research on the default mode network, for example, reveals that performing focused tasks reduces hippocampal activity relative to the default state with no task^26^, and such reductions may impair memory. It is noteworthy, however, that we did not observe amnesia from other complex tasks that did not require suppression (for example, memory retrieval, updating and phonological rehearsal—buffered by odd/even judgments), suggesting that memory disruption is specific to retrieval suppression processes and not to blood–oxygen-level dependent reductions more broadly. Exploring such possibilities in conjunction with neuroimaging designs adapted to track bystander-related activity during and after their presentation would complement the present focus on behavioural sequelae of suppression-related modulations. Regardless of whether the amnesic shadow is unique to suppression, what is clear is that suppressing hippocampal activity through cognitive control induces a state akin to a virtual lesion, mimicking hippocampal amnesia.

Cognitively induced amnesia carries mechanistic implications for retrieval suppression and cognitive control more broadly. Retrieval suppression is known to harm retention of the suppressed memories, but prior behavioural and neuroimaging work did not address whether inhibitory control mechanisms targeted specific memory traces or hippocampal retrieval processes more systemically. The present evidence, however, permits clear inferences about functional breadth of the suppression mechanism: only a broad, systemic suppression mechanism predicts that suppressing retrieval would cause forgetting of diverse stimuli (words, objects and scenes) that were entirely unrelated to the suppressed content. Critically, this systemic targeting not only interrupts pattern completion, but also affects encoding and stabilization processes needed by other memories. If so, this finding holds general lessons about cognitive control. It implicates a class of inhibitory control mechanisms differing from biased competition^27^—a class that relies instead on direct systemic inhibitory modulation to suppress regional processing^28^,^29^. Thus, systemic suppression may be not only a key mechanism of memory control, but also a general principle of inhibitory control that is relevant to many other domains, such as affect regulation and motor stopping.

The present phenomenon reveals a force affecting memory for life experiences that appears unexplained by current theories of forgetting. Over the last century, forgetting has been attributed to decay, interference^30^, contextual change between encoding and retrieval^31^, inhibition^32^ and disrupted hippocampal consolidation arising from the encoding of subsequent interfering memories^33^,^34^. Memories lost to the amnesic shadow appear not to be forgotten for these reasons; compared with control (zero dose) items, they are equally old and subject to decay; they are preceded and followed by the same bystanders, equating interference; and they are not direct targets of inhibitory control. Moreover, although we have argued for interrupted memory stabilization, here disruption arises from suppression and not merely from generic intervening mnemonic activity^14^,^35^. Indeed, although ample precedent exists suggesting that the mnemonic activity that intervenes between initial presentation and final test of bystanders should harm their retention through inteference^35^, the total amount of this interfering activity was matched across zero- and two-dose conditions in all current studies. The additional forgetting arising from adjacent suppression trials can, therefore, be isolated to the disruptive effects of suppression, as indicated by experiments 3–5. Suppression appears to disrupt processes following event encoding that are necessary to stabilize memories, though it remains unclear whether it only disrupts input to consolidation mechanisms, or also the consolidation process itself. Regardless of the particular process affected, our findings suggest that hippocampal downregulation—whether achieved via retrieval suppression or other task states—is a potent force unaddressed by classical or modern theories of forgetting.

Identifying this forgetting mechanism is not merely of theoretical concern. Evidence suggests that it may sometimes underlie profound memory deficits. After major traumas, intrusive memories pervade experience, and people often cope by trying to prevent their retrieval. If so, systemic suppression of hippocampal activity may punctuate experience to such a degree that amnesic shadows become debilitating. Indeed, memory deficits often accompany acute and post-traumatic stress disorders, even for neutral memories unrelated to the trauma^36^,^37^,^38^. Hippocampus-dependent tasks such as associative recognition are impaired dramatically, with minor item recognition deficits (as observed here)^38^. Strikingly, memory problems in these populations often dissipate as intrusions abate^37^. These deficits have received much attention^36^ with explanations focusing on trauma-related factors such as distractibility^39^, poor sleep^40^ and stress-related neurochemicals that trigger hippocampal dysfunction^41^. Our findings suggest another complementary factor. They highlight an arrestingly simple dynamic that may contribute to the cruel irony of being haunted by traumatic memories while suffering impoverished retention of daily events: Efforts to forget a troubling past may, ironically, leave amnesia for the present in their wake.

## Methods

### Overview of basic methodology

A modified TNT protocol^5^,^42^ was used. Participants initially learned cue-target word pairs to criterion (50% minimum in experiments 2, 4 and 5; 100% in the remaining experiments). During the critical TNT phase that followed, cues were presented in isolation, with the display colour indicating whether the target should be silently retrieved (if green) or suppressed (if red). Cues were repeated 12 times, except for the third reserved as baseline items, which were not represented during the TNT phase.

Between Think and No-Think trials, participants occasionally encountered novel ‘bystander' stimuli that were unrelated to the word pairs, for which they completed an orienting task. In experiments 1–5, the orienting task was to silently generate an explanation for why the central object in the bystander photograph (for example, peacock) appeared in the pictured background (for example, parking lot) and then to rate the difficulty in generating that explanation on a four-point scale by pressing a button (1=‘no difficulty' to 4=‘extreme difficulty'). Experiments 6 and 7 asked participants to judge whether the (written or pictured) object presented on that trial was animate or pleasant, depending on the visual instruction prompt. Bounding every critical bystander—and in experiment 7, all trial types—was a block of odd/even buffer judgments. During buffer periods, participants decided whether each of a sequence of digits (experiments 1, 3 and 6) or the sum of two digits (experiments 2, 4, 5 and 7) was even by pressing the appropriate button as quickly and as accurately as possible. These buffers were included to match the local task shifts into and out of the bystander presentations across conditions.

Most experiments required participants to suppress associates of red cues directly—that is, without recalling substitute thoughts or memories that might engage hippocampus-dependent retrieval processes. Experiment 3 pitted these strategies (direct suppression and thought substitution) against each other, and strategy choice was left to participants' discretion in elements of experiment 6. In all cases, they were to fully attend to and fixate on the presented TNT cues. Participants' understanding of and adherence to task instructions, including those related to direct suppression, were assessed via diagnostic questionnaires administered throughout the practice sessions before the TNT phase and also halfway through the TNT session. After the experiment, participants also rated their compliance with the No-Think instructions.

In between the TNT phase and the post-experiment questionnaire, participants' memory for bystander items was tested, as detailed in the body of the report. The object-recall test in experiments 1–5 had a timeout of 15 s. The two-step recognition test in experiment 6 first presented a six-point confidence scale for old/new item recognition before a context memory judgment about whether the bystander was originally presented as part of the animacy or pleasantness task (Supplementary Fig. 1). Experiment 7 only tested context memory. Before the post-experiment questionnaire, participants' memory for TNT associates was tested (Supplementary Methods and Supplementary Data).

### General description of participants

The number of participants per experiment varied, primarily due to counterbalancing constraints. Participants were also excluded if they did not meet eligibility requirements or evidenced a persistent and purposeful failure to comply with suppression instructions^43^, as assessed by a post-experiment questionnaire (see Supplementary Table 1 for exclusions and further details). The final sample, across experiments, involved 381 unique participants ranging in age from 18 to 35 years, 245 of whom were female.

Participants were recruited from three sites (the University of Oregon (Eugene, Oregon, USA), the University of St. Andrews (St. Andrews, Scotland), and the Medical Research Council's Cognition and Brain Sciences Unit (MRC–CBU, Cambridge, England)) purportedly to partake in an experiment designed to assess their ability to pay attention and ignore distracting things. Experiment protocols were approved by the relevant ethics committee (Oregon: Institutional Review Board protocol #C1-314-07F; St Andrews: University Teaching and Research Ethics Committee reference number PS5008; MRC–CBU: Cambridge Psychology Research Ethics Committee reference number 2009.60). Written informed consent was obtained from all participants. See also Supplementary Table 1.

Eligible participants were neurologically healthy without diagnosed learning/reading/attention disorders, had normal/normal-corrected colour vision, and were exposed to English as a primary language since early childhood (0–3 years). Additional constraints were instituted according to the operational standards at the MRC–CBU for the fMRI experiment. Participants were to be right-handed and not have metal in their bodies, be claustrophobic, or be on psychoactive medications. Participants were asked to sleep a minimum of 6 h the night before their session(s). Participants were to have had no previous experience with the TNT protocol. Remuneration came in the form of payment or—in the cases of the Universities of Oregon and St. Andrews—credit towards fulfilling a requirement for an introductory psychology course.

### General materials

There were 36 critical TNT word pairs and a variable number of fillers. The nature of the stimuli and many of the pairs themselves followed previous work^5^. Although the stimuli were constructed under the same stipulations, experiment 7 only called for 30 critical TNT pairs (10/condition). The constituent members of each pair (for example, ‘LEAP-BALLET') were designed to be associable but not so associated as to encourage a reliance on guessing. Word-association norms helped guide and validate stimulus selection^44^. Care was taken to avoid pre-existing semantic relationships between items from different TNT pairs, with the bystanders and with TNT test cues. TNT associates (for example, ‘BALLET') were selected such that each was an exemplar of a unique superordinate category (for example, ‘DANCE'), with which recall could be cued on the independent-probe test, taking the form of ‘DANCE-B___.' This constraint was relaxed in experiment 4 because no independent-probe test was administered. This test assesses the accessibility of a target memory itself, independent of the strength of the original cue-target association^32^,^42^, whereas the same-probe test cues recall with the learned cue (e.g., ‘LEAP').

Critical TNT word pairs were randomly divided into three subsets and counterbalanced across participants, as a rule. One-third of the cues would appear in a green rectangle during the TNT phase (Think cues). Another third would appear in a red rectangle (No-Think cues). The remaining third of the items (Baseline) did not reappear during the TNT phase. Participants at Oregon in experiment 6 followed a similar protocol; however, the cue words were presented in a diagnostic red/green font, without a surrounding rectangle.

Participants in most experiments were provided with brief precues and postcues surrounding every Think and No-Think trial (Supplementary Fig. 1). The precues were designed to facilitate preparation of the relevant task set^45^. Displays during these periods consisted of a lone rectangle—the same size and colour in which the TNT cue was presented. As such, participants first saw a coloured rectangle for 750 ms, onto which a TNT cue was superimposed for 2500, ms before it disappeared, leaving a blank, coloured rectangle for an additional 250 ms (see Supplementary Methods and Supplementary Table 1 for timing variations).

Critical bystanders in experiments 1–5 consisted of 32 digital photographs (and 27 fillers) of real-world backgrounds with and without a distinctive, nameable central object present. Each posed still was captured from the same vantage point twice: once with a nameable central object and once with the central object missing. The background settings and objects were chosen to be relatively distinct. For the word recognition variant of experiment 6, a set of 64 critical nouns (half to serve as targets and half as lures in the subsequent item recognition test) was drawn from the MRC's psycholinguistic database^46^, with preference for words from an earlier neuroimaging study of item and context memory^20^. Roughly half of the critical items represented something living, with the remainder being non-living according to norms and the experimenters. For the picture recognition variant of experiment 6, the bystander words described above were substituted with the same number of full-colour photographs (often pictorial representations of the same nouns against a white background). These stimuli were selected to limit semantic clustering. Experiment 7's stimulus set was based on the word version of experiment 6, but it was expanded to include 78 critical target nouns. As we did not test item memory in experiment 7, no lures were held in reserve.

A visual warning preceded bystander trials. In experiments 1–5, the prompt ‘object/setting' appeared centrally for 500 ms and was then immediately replaced by the photo and rating scale. Experiments 6 and 7 similarly incorporated the prompts ‘living?' and ‘pleasant?' Before the first odd/even judgment in a series, participants received a warning in the form of the prompt ‘even?' for 500 ms across all experiments, with the exception of those participating in Oregon for experiment 6, who received a central fixation cross instead lasting 300 ms.

The four possible permutations of Think and No-Think trials surrounding bystanders were uniformly distributed across the entire TNT phase, as well as within each of the eight (or six, in the case of experiment 7) constituent blocks. At a minimum, the first and last four TNT trials—as well as the first and last bystanders—in each block were fillers so as to control for primacy and recency effects; however, for imaging purposes, experiment 7 did not present any filler items (TNT or bystanders) during the critical TNT phase. Otherwise, block randomization was used to create the presentation schedules, with filler items distributed to minimize the predictability of the upcoming trial type. Manual alterations to the randomization were occasionally introduced to ensure all of the relevant bystander-TNT permutations of interest occurred in each block.

The assignment of bystanders to these four permutations (that is, the TNT frame, which could consist of no suppression either before or after the bystander, a single suppression epoch before the bystander, a single suppression epoch after the bystander or a suppression epoch both before and after the bystander) was counterbalanced across participants, except in experiment 7, for which item assignment was automatically randomized for each participant. The type of judgment performed on the bystander (that is, living or pleasantness judgment) was similarly randomized for each item in experiment 7.

In experiments 1, 3 and 6, a pre-determined series of two to four odd/even buffer judgments was presented around critical bystanders during the TNT phase. After a response or a timeout (at 900 ms), the next trial was presented. In the other experiments, participants had a fixed period of time (6500, ms in experiments 2, 4 and 5; a jittered duration in experiment 7) to complete as many semi-self-paced odd/even judgments as possible on the sums of successively presented pairs of digits. Participants were instructed to make these judgments as quickly and as accurately as possible.

### General analysis approach

The Greenhouse–Geisser correction was applied when a potential violation of the sphericity assumption was detected by Mauchly's test. An alpha level of 0.05 was used throughout, and estimated marginal means are provided for descriptive purposes. Also for the purposes of visualization, plots intended to compare within-participant variance incorporate error bars derived from a method that first standardizes the data across participants^47^. Difference scores and between-participant effects are plotted with error bars reflecting traditional s.e.m.

In computing accuracy scores across measures of interest, non-responses, while rare, were coded as incorrect. Reaction time statistics were computed on the basis of correct trials only, when accuracy was determinable. Although the raw reaction times are reported for display purposes, statistical analyses were performed on the base-10 log-transformed data to normalize the positive skew that often arises with zero-gated reaction time measures. Transforming the data in this way did not meaningfully change the conclusions drawn from parallel analyses on the raw data.

We largely focused our analyses on bystander events (and surrounding odd/even judgments) presented in the second half of the TNT phase, after participants had received ample practice at retrieval suppression. This focus was based on prior published work reporting suppression-related hippocampal deflections from pretrial baseline activity only after sufficient suppression practise^6^. To improve statistical power in the experiment 7, we had participants practise retrieving/suppressing the TNT word pairs before introducing any critical bystanders. As such, we analysed bystander recall irrespective of their placement in the TNT phase (follow-up analyses supported the assumption that there were no differences across halves).

### Bystander cued recall

Cued-recall responses for bystander objects were coded for accuracy and entered into a mixed-design analysis of variance, with the number of surrounding suppression epochs (0, 1 or 2) entered as a repeated measures factor, along with the half into which the bystander item was introduced during the TNT phase. Coding was done blind to condition. A similar analysis approach was taken for the other bystander/online measures. Item counterbalancing was included as a between-participant factor. Planned contrasts allowed for tests of the amnesic shadow effect, defined as the difference in cued-recall accuracy between the zero- and two-epoch conditions, for bystanders presented in the second half of the relevant behavioural experiment.

### Bystander item recognition

Responses along the six-point confidence scale for item recognition, which ranged from ‘1' (definitely new) to ‘6' (definitely old), were divided into two bins representing items the participant judged as ‘new' (1–3 responses) or ‘old' (4–6 responses). Accuracy was computed by comparing the reported status (old or new) to veridical status (target or foil). As participants were informed that they should focus on accuracy and not be rushed, accuracy—rather than reaction time—served as the dependent measure.

### Bystander context recognition

Participants were again encouraged to respond as carefully and accurately as possible, having been provided ample time to do so. As above, accuracy was the dependent measure.

### Online measures of attention

Accuracy and reaction time on the odd/even buffer tasks were analysed based on whether the relevant decisions followed a Think or a No-Think trial. Participants' subjective ratings (on the four-point difficulty scale) to the bystander orienting task in experiments 1–5 were analysed on the basis of number of surrounding suppression epochs. Reaction times were analysed in a similar manner, though participants were not pressured to respond quickly, so long as they responded within the allotted 5 s. Responses to the living and pleasantness judgments in experiment 6 and 7 were registered using ‘yes' and ‘no' buttons. Participants were instructed to use intuition and not linger over their decision, should the cue be considered ambiguous with respect to the orienting task. Results for each behavioural experiment are presented in Supplementary Table 2.

### TNT final test data

To reduce noise in the final test measures owing to differences in learning, accuracy was computed conditionalized on successful recall of a given item during the final round of testing during the initial learning phase. Conditionalized accuracy on the TNT final tests was entered into a mixed-design analysis of variance, with condition (Think, No-Think, and Baseline) entered as a within-participant factor and item counterbalancing as a between-participant factor. Planned contrasts isolated the negative- and positive-control effects, measured as differences from Baseline recall for No-Think and Think conditions, respectively.

### Data availability

Imaging data (MR10010) are stored on the MRC–CBU archive and are available on request.

## Additional information

**How to cite this article:** Hulbert, J. C. *et al*. Inducing amnesia through systemic suppression. *Nat. Commun.* 7:11003 doi: 10.1038/ncomms11003 (2016).

## Supplementary Material

Supplementary Information Supplementary Figures 1-4, Supplementary Tables 1 and 2, Supplementary Notes, Supplementary Methods and Supplementary References.
