# Supplementary material for: Inducing amnesia through systemic suppression
Source: Nat Commun. 2016 Mar 15;7:11003. doi: 10.1038/ncomms11003 (PMC4796356; doi:10.1038/ncomms11003)
Supplement: Supplementary Information — Supplementary Figures 1-4, Supplementary Tables 1 and 2, Supplementary Notes, Supplementary Methods and Supplementary References. [file ncomms11003-s1.pdf]

SUPPLEMENTARY FIGURES:

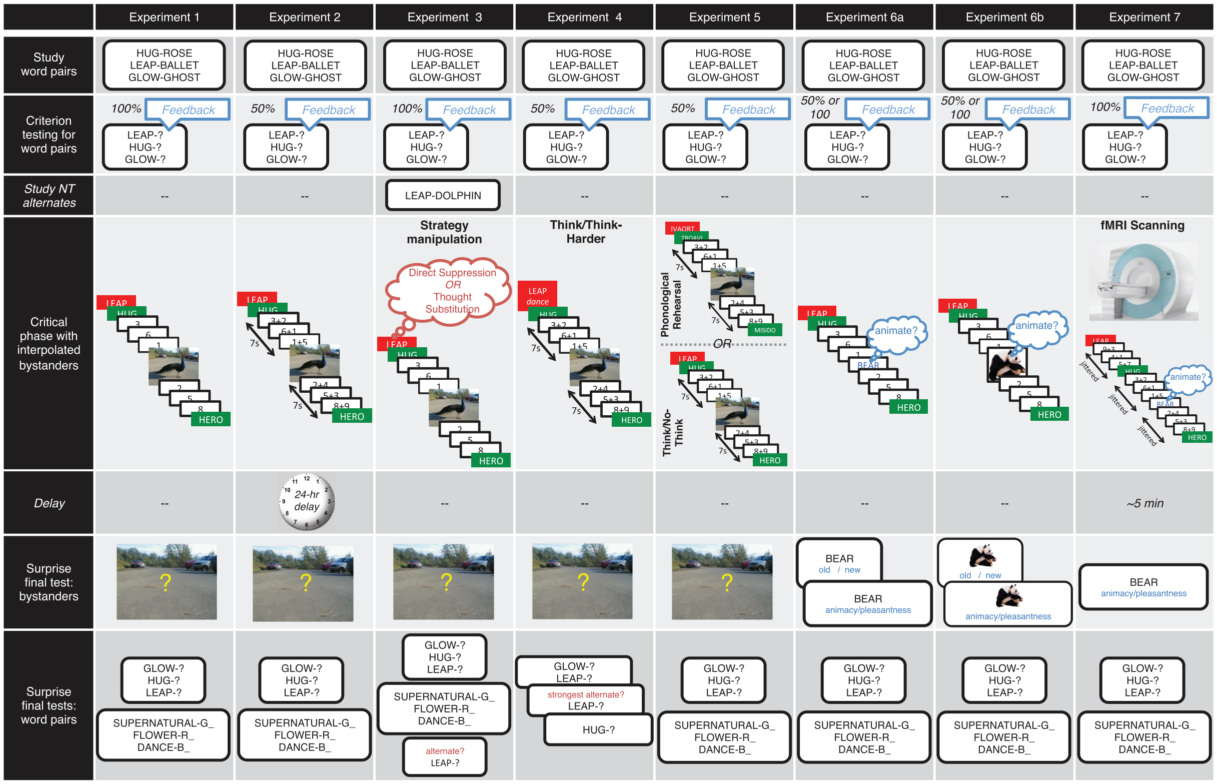

Supplementary Fig. 1 | Overview of the paradigm used in experiments 1-7, with representative examples. In the most basic form, the experiment required participants to learn the TNT pairs to criterion (either 50% or 100%), engage in the modified TNT ("H.M.") phase with interpolated bystanders and odd/even buffer judgments, and participate in a surprise memory test for aspects of the bystander materials. A final test for the learned TNT associates followed. See Fig. 1, Methods, and Supplementary Notes for further details.

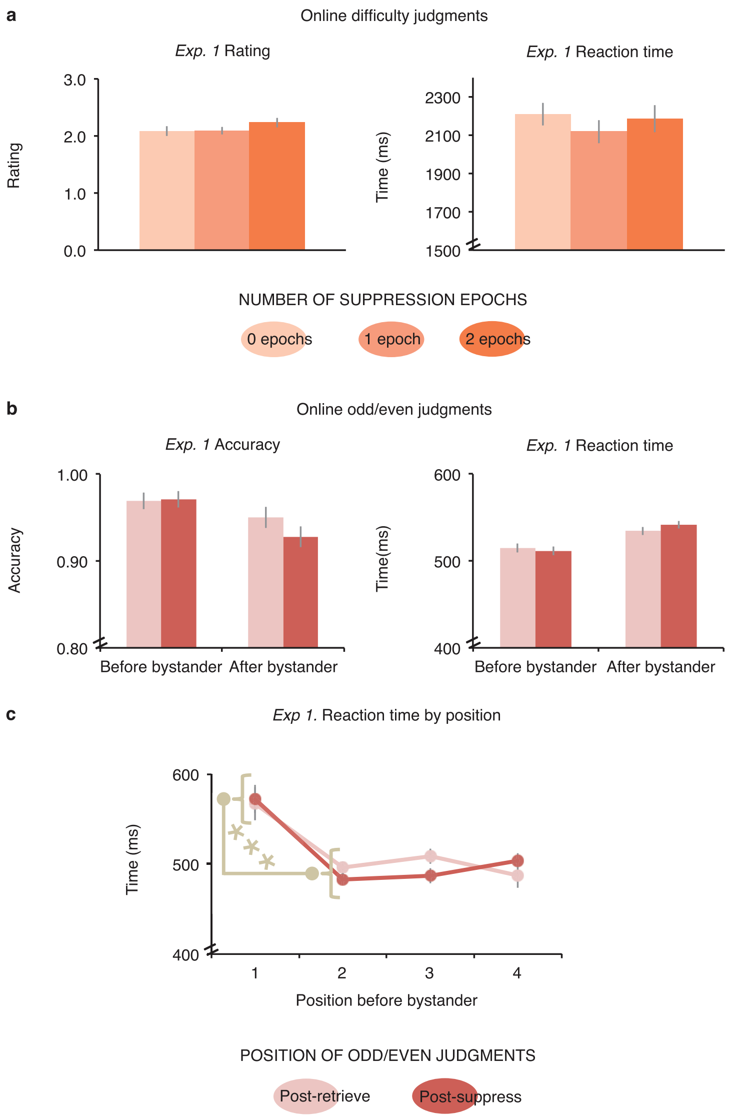

Supplementary Fig. 2 | Attention and task switching. a, Ratings and reaction times during experiment 1's orienting task (subjective difficulty of explaining objects' placement). **b,** Accuracy and reaction times for the odd/even buffer judgments occurring before (left pairs) and after (right pairs) bystander presentation reveal no reliable differences between post-retrieval (pink) and post-suppression (red) judgments. **c,** Mean reaction times for buffers by serial position after the TNT trial/before the bystander. Slower initial reaction times (versus positions 2-4) indicate an overall switch cost when participants transitioned from a Think/No-Think trial that leveled off at longer lags. Error bars reflect within-participant s.e.m. (N=24). Statistical comparisons reflect results of F-tests; ***P<0.001.

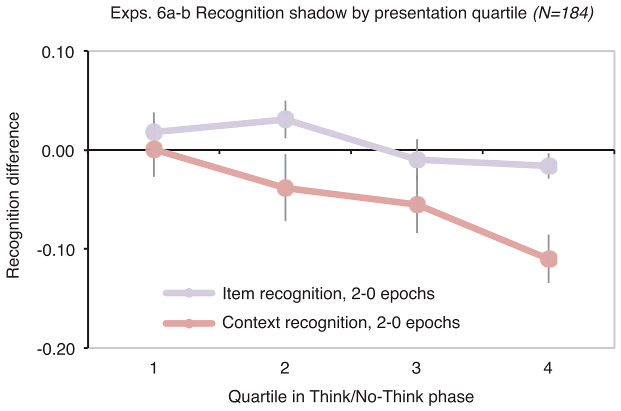

Supplementary Fig. 3 | Growth of the amnesic shadow in recognition memory. The amnesic shadow in context recognition grew across quartiles of the TNT phase, much like it did in cued recall. In contrast, the shadow was absent in all blocks for item recognition. Error bars reflect s.e.m.

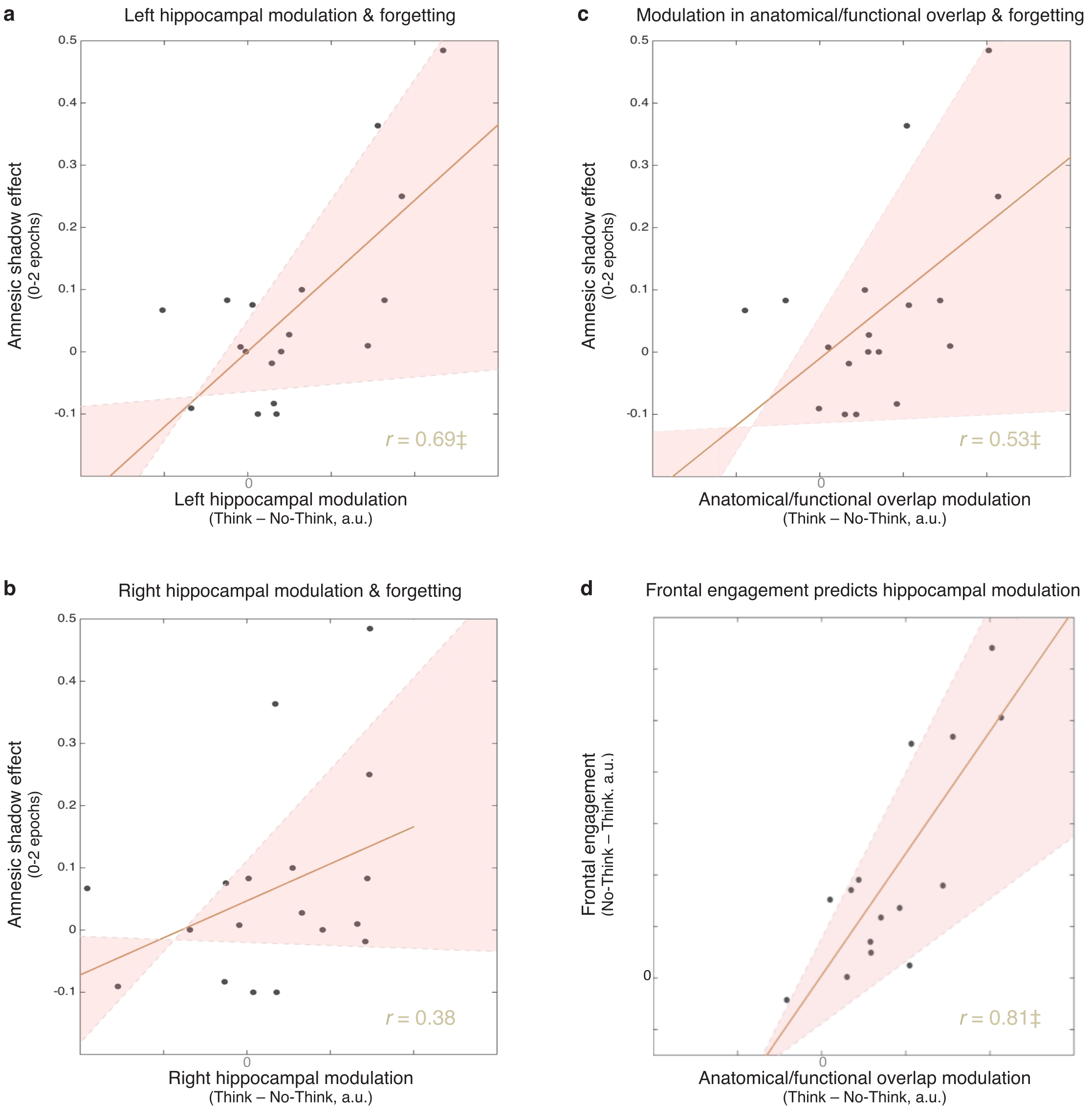

Supplementary Fig. 4 | Amnesic shadow and hippocampal modulation. a, Results of an exploratory analysis revealing individual differences in modulation from anatomically defined left hippocampal ROI positively correlated with context amnesia (outlier-skipped Pearson bootstrap 95% c.i.: [0.13 0.88]). **b,** A similar, though non-significant, trend for the anatomically defined right hippocampus (bootstrap c.i.: [-0.04 0.67]. **c,** Modulation in the portion of the (left) hippocampus overlapping with the whole-brain contrast reliably predicted amnesia (bootstrap c.i.: [0.07 0.79]). **d,** Suppression-related activation in right lateral prefrontal cortex further predicted modulation within this overlap (bootstrap c.i.: [0.46 0.92]). Red bands represent 95% c.i.; ‡significant correlation according to bootstrap test.

SUPPLEMENTARY TABLES:

	Experiment							
	1	2	3	4	5	6a	6b	7
Site	SA	CBU	SA	CBU	CBU	SA/UO	SA/UO	CBU
Learning criterion	100%	50%	100%	50%	50%	100%/ 50%	100%/ 50%	100%
Suppression instructions	DS	DS	DS vs. TS	TH	DS vs. Ph	DS/GS	DS/GS	DS
Odd/even task	Digit	Sum	Digit	Sum	Sum	Digit	Digit	Sum
TNT pre- & postcues	Yes	Yes	Yes	Yes	Yes	Yes/No	Yes/No	Yes
TNT cue word duration (ms)	2500	2500	2500	4†	2500	2500/ 2350	2500/ 2350	3000
Final sample size (# males)	24(9)	24(10)	24(9) vs. 24(5)	36(15)	24(5) vs. 23(9)	24(4)/ 48(20)	48(19)/ 64(22)	18(9)
**Exclusions**								
Failed to reach criterion	--	2	--	--	3 vs. 0	--	--	--
No-show (time 2)	--	1	--	--	--	--	--	--
TNT non-compliance	1	5	2 vs. 0	--	--	1/0	2/2	1
Lack of sleep	--	--	--	--	--	--	3/0	--
ADHD	--	--	--	--	0 vs. 1‡	0/1	--	--
Fell asleep	--	--	--	--	--	--	--	2
Claustrophobia	--	--	--	--	--	--	--	1
Bystander recall at floor	--	--	--	1	--	--	--	--
Dyslexia	1	--	--	--	--	--	--	--
Brain damage	--	--	--	--	--	--	2/0	--

Supplementary Table 1 | Participant and methodological details, by experiment. SA = University of St. Andrews; CBU = Medical Research Council's Cognition and Brain Sciences Unit; UO = University of Oregon; DS = Direct suppression; TS = Thought substitution; TH = Think-Harder task; Ph = Phonological rehearsal; GS = Generic suppression instructions; † = Semi-self-paced trial timing; ADHD = Attention deficit/hyperactivity disorder; ‡ = No replacement run for this condition.

Experiment		0 epochs	2 epochs	*P*-value	*N*
1 – Basic shadow effect					
	Buffer RT (s)	0.530 (0.01)	0.527 (0.01)	0.691	24
	Buffer acc.	0.948 (0.01)	0.948 (0.01)	1.000	24
	Bystander RT (s)	2.21 (0.16)	2.18 (0.16)	0.712	23
	Bystander rating	2.08 (0.18)	2.23 (0.16)	0.275	23
2 – Shadow after 24hrs					
	Buffer RT (s)	0.889 (0.04)	0.893 (0.04)	0.893	24
	Buffer acc.	0.914 (0.02)	0.912 (0.02)	0.842	24
	Bystander RT (s)	2.56 (0.13)	2.60 (0.17)	0.882	23
	Bystander rating	1.78 (0.13)	1.79 (0.14)	0.944	23
3 – Strategy manipulation *(direct suppression group)*					
	Buffer RT (s)	0.538 (0.01)	0.535 (0.01)	0.513	24
	Buffer acc.	0.910 (0.02)	0.924 (0.02)	0.471	24
	Bystander RT (s)	1.92 (0.15)	1.98 (0.12)	0.293	24
	Bystander rating	1.95 (0.15)	2.00 (0.15)	0.726	24
3 – Strategy manipulation *(thought substitution group)*					
	Buffer RT (s)	0.536 (0.01)	0.534 (0.01)	0.721	24
	Buffer acc.	0.924 (0.01)	0.917 (0.02)	0.655	24
	Bystander RT (s)	2.33 (0.18)	2.33 (0.17)	0.674	24
	Bystander rating	1.93 (0.12)	1.95 (0.15)	0.879	24
4 – Difficulty manipulation					
	Buffer RT (s)	0.873 (0.03)	0.870 (0.03)	0.988	36
	Buffer acc.	0.924 (0.01)	0.921 (0.01)	0.737	36
	Bystander RT (s)	2.77 (0.11)	2.94 (0.11)	0.104	36
	Bystander rating	1.98 (0.10)	2.13 (0.10)	0.191	36
5 – Components of the shadow *(direct suppression group)*					
	Buffer RT (s)	0.904 (0.03)	0.918 (0.04)	0.425	24
	Buffer acc.	0.918 (0.01)	0.903 (0.02)	0.169	24
	Bystander RT (s)	2.45 (0.16)	2.58 (0.14)	0.169	24
	Bystander rating	1.79 (0.10)	2.21 (0.14)	0.003**	24
5 – Components of the shadow *(baseline group)*					
	Buffer RT (s)	0.957 (0.04)	0.956 (0.04)	0.881	23
	Buffer acc.	0.911 (0.02)	0.924 (0.01)	0.265	23
	Bystander RT (s)	2.74 (0.14)	3.02 (0.17)	0.058	23
	Bystander rating	1.99 (0.13)	2.05 (0.12)	0.606	23
6a – Shadow in recognition memory *(words)*					
	Buffer RT (s)	0.513 (0.01)	0.510 (0.01)	0.215	72
	Buffer acc.	0.933 (0.01)	0.940 (0.01)	0.303	72
	Semantic RT (s)	0.754 (0.02)	0.756 (0.02)	0.921	67
6b – Shadow in recognition memory *(pictures)*					
	Buffer RT (s)	0.516 (0.01)	0.512 (0.00)	0.070	111
	Buffer acc.	0.936 (0.01)	0.938 (0.01)	0.761	111
	Semantic RT	0.723 (0.02)	0.714 (0.02)	0.647	111

Supplementary Table 2 | Measures of attention by experiment. Means (traditional s.e.m.) for judgments pertaining to critical bystander items presented in the latter half of the TNT phase for the 0- and 2-epoch conditions, with associated *P*-values reflect *F*-tests contrasting them. Rightmost column indicates the number of valid (non-empty) cases tested. RT = reaction time; Acc. = accuracy; Buffer = odd/even buffers surrounding bystanders; Bystander rating = subjective difficulty rating given to the bystander orienting task (generating an explanation for why the central object appears in the given background); Semantic = the animacy/pleasantness orienting task in recognition studies; ***P*<0.01.

SUPPLEMENTARY NOTES:

Experiment 2

After the second session, participants were asked about whether they suspected a final test for the bystander items [‘0’ (not at all) to ‘4’ (definitely suspected a test)] and the degree to which they rehearsed the materials spontaneously or intentionally [‘0’ (never) to ‘4’ (very frequently)] over the 24-hour delay. The mean suspicion score was 0.13 (SD = 0.45, range = 0-2). Participants indicated (using the latter scale) that they were only rarely spontaneously reminded of their bystanders during the delay (M = 0.21, SD = 0.59, range = 0-2). According to their report, they hardly ever attempted to bring a bystander to mind or quizzed themselves on the bystanders during the delay to prepare for a possible later test (M = 0.04, SD = 0.20, range = 0-1). None of these measures correlated with the amnesic shadow effect.

In addition to the overall analyses reported in the main text, we examined whether the anterograde and retrograde shadows varied as a function of immediate versus delayed testing. We compared these effects in experiment 2 (24-hour delay) versus all immediate recall studies. For this particular analysis, a single outlier participant was removed from experiment 2 (> 2.5 standard deviations away from mean effect and 0% recall in all but one cell; all analyses remain significant with outlier included). In the remaining sample of 95 participants, the amnesic shadow was reliable across all recall studies, both for the anterograde effect (0 suppression doses versus 1 suppression dose preceding the bystander), $F_{1,79} = 30.63$, $P < 0.001$, and the retrograde effect (0 suppression doses versus 1 suppression dose following a bystander), $F_{1,79} = 7.82$, P = 0.006. Neither effect interacted with delay (anterograde and retrograde effects by delay, $F < 1$ in each case). These findings indicate that shadow effects were not reliably altered by delay.

Experiment 4

T/TH final recall. Conditionalized recall of the initial T/TH associates was high overall, with no effect of condition ($F_{2,66} = 1.34$, $P = 0.269$). Recall of the strongest alternate for each critical Think-Harder cue (i.e., the strongest/last alternate they switched to) was comparatively low ($M = 0.56$, $SD = 0.27$); this is not necessarily surprising given the interference generated by all of the alternates introduced throughout.

Correlations between T/TH recall and the amnesic shadow. As reported elsewhere, neither the amnesic shadow effect (here defined as the difference in recallability between bystanders from the latter half of the presentation schedule that were surrounded by two Think-Harder trials compared to two Think trials) nor the difference in the recall between Think-Harder and Baseline associates was significant. There was no significant correlation between these two measures (Pearson skipped $r = -0.05$, 95% bootstrap c.i. = [-0.34 0.27]).

Stay/switch accuracy. Participants were nearly perfect ($M = 0.97$, $SD = 0.11$) at pressing the appropriate button on Think trials, as well as being fast ($M = 1105$ms, $SD = 230$), considering trials from the relevant second half of the presentation schedule. One indication that the Think-Harder trials were, indeed, more difficult arose from the relative reduction in accuracy [$M_{Think\text{-}Harder} = 0.89$, $SD = 0.05$; $F_{1,33} = 25.61$, $P < 0.001$] and increase in reaction time [$M_{Think\text{-}Harder} = 1648$ms, $SD = 374$; $F_{1,33} = 107.85$, $P < 0.001$]. Considering that the stay/switch decisions for the Think-Harder trials were somewhat subjective, the high accuracy indicated that participants were, largely, on task. Neither the difference between Think and Think-Harder performance on stay/switch accuracy (skipped Pearson $r = 0.16$, 95% bootstrap c.i.; [-0.18 0.45]) nor the same contrast performed on log-transformed reaction times (skipped Pearson $r = 0.17$, 95% bootstrap c.i.: [-0.14 0.48]) correlated with the shadow effect.

Experiment 5

Bystander recall. As expected, bystander recall in the phonological rehearsal group did not differ by the type of surrounding baseline task (forwards or backwards rehearsal), $F < 1$. Therefore, for our baseline, we collapsed across these conditions.

Relative to the baseline, conditions in which suppression followed bystander presentation yielded reliably worse recall ($F_{1,39} = 4.97$, $P = 0.032$), whereas conditions in which suppression preceded bystander presentation yielded marginally significant impairment, $F_{1,39} = 3.16$, $P = 0.083$.

Experiment 6

Growth of the amnesic shadow in recognition memory. As reported elsewhere, the context memory deficit in recognition grew linearly over quartiles of the Think/No-Think phase (Supplementary Fig. 3; $F_{1,152} = 6.65$, $P = 0.011$); however, no linear effect of quartile emerged for item memory ($F_{1,152} = 0.143$, $P = 0.706$), dissociating the effect of systemic suppression on these two measures.

Analysis of buffer task data relevant to task switching

The overall analysis of buffer task data across behavioral TNT experiments ($N = 279$; see Supplementary Table 2) indicates that participants did not exhibit a reliable difference in buffer trial response time following suppression and retrieval tasks ($F < 1$). This lack of difference across conditions arose despite clear evidence that the buffer task (overall) was sensitive to the distracting effects of task-set switching: When reaction times were broken down by the serial position of a buffer trial, they were considerably longer for the first buffer trial that occurred immediately after a TNT trial ($M = 667$ms), compared to the remaining trials ($M = 590$ms; $F_{1,270}$

= 269.19, $P < 0.001$; M for positions 2, 3, and 4 = 585ms, 596ms, 590ms, respectively). Buffer task accuracy followed a similar pattern with the first buffer trial following a TNT trial (M = 0.930) tending (non-significantly) to be responded to less accurately than the remainder (M = 0.944; $F_{1,271} = 3.63$, $P = 0.058$). Thus, despite clear evidence of switch cost effects, no overall difference in response efficiency was found.

To scrutinize this issue further, we examined whether the reaction times on the very first trial following a TNT task differed depending on whether subjects had just performed a retrieval or suppression trial. With this focused analysis, evidence for a difference across conditions was indeed obtained, with reaction times on the first buffer trial being reliably longer following suppression (M = 674ms) than following retrieval (M = 659ms; $F_{1,270} = 8.16$, $P = 0.005$). Moreover, the switch cost, as operationally defined by the difference between the first and second trials, was reliably larger following suppression (M = 97ms) than following retrieval (M = 67ms; $F_{1,270} = 27.82$, $P < 0.001$). This finding illustrates why including the buffer task was an important procedural control, in order to dissipate differential distraction due to switching between tasks. To confirm that the switch cost indeed dissipated thereafter, we examined reaction times following the first trial and found no reliable differences (M = 589ms for post-suppression; M = 591ms for post-retrieval; $F_{1,270} = 0.957$, $P = 0.329$). Accuracy also was high in the two conditions and did not vary reliably in any of the four buffer trials following a TNT trial, except on the fourth trial, $F_{1,271} = 6.82$, $P = 0.010$. However, in this case, accuracy was reliably higher following suppression ($M = 0.952$) than following retrieval ($M = 0.903$), contrary to the notion that suppression was more distracting.

Taken together, these findings indicate that the buffer task was sensitive to distraction due to task switching, in general, and confirmed concerns about differential task demands that

motivated inclusion of the buffer task. Importantly, they also showed that the small but reliable effect of differential distraction dissipated rapidly, as suggested by the absence of any reliable difference in overall performance on the buffer task.

Mega-analysis of final Think/No-Think recall data across behavioral studies

Across the 280 participants who received standard TNT instructions, suppression items were recalled more poorly than were baseline items on conditionalized final recall ($F_{1,264}$ = 23.63, $P < 0.001$). Think associates, however, were not reliably facilitated above baseline ($F_{1,264}$ = 3.42, $P = 0.066$).

Experiment 7

Behavior. A full round of retrieval/suppression practice using the critical TNT items prior to the actual TNT phase was added prior to the fMRI data acquisition phase. This was motivated by the strong tendency for amnesic shadows to develop over blocks; adding extended practice prior to scanning enabled us to use the behavioral and imaging data from the entire scan session, rather than only from the second half. As such, we analyzed context memory recognition accuracy from bystanders presented throughout the entire TNT phase (as expected, there weren't interactions by the half of the TNT phase into which a bystander was introduced, *F*s < 1).

Overall context recognition was not reliably impaired for bystanders in the 2-epoch condition, relative to the 0-epoch condition ($F_{1,17}$ = 0.78, P = 0.389), with an average 2.6% amnesic shadow effect across participants. This muted overall behavioral effect may have arisen from the much longer runs of odd/even trials (lasting as long as 22s on either side of the bystander, compared to an average of 5.2s across experiments 1-6). Nevertheless, the overall recognition shadow ranged from a 14% reversal to a 33% result in the predicted direction across

participants, and these individual behavioral differences reliably correlated with degree of modulation in the bilateral hippocampus (skipped Pearson r = 0.57, 95% bootstrap c.i.: [0.11 0.82]), as well as in our follow-up exploratory examination of the left hippocampus (skipped Pearson r = 0.66, 95% bootstrap c.i.: [0.16 0.87]).

These brain-behavior relationships remained unchanged when we restricted our analysis to only those bystanders whose average odd/even buffer durations were matched to the buffer durations used in our six behavioral experiments (see Fig. 4d-e and Supplementary Fig. 4). When considering just these buffer-delay matched items, the context recognition shadow was quantitatively similar (M = 6.4%) to that observed in experiment 6 (M = 8%) and revealed a trend towards the predicted amnesic shadow ($F_{1,17}$ = 2.99, P = 0.10, Fig. 4c). Analyses confirmed that the recognition shadows did not differ reliably across experiments 6 and 7 when buffer durations were matched (interaction of shadows across experiments, $F < 1$).

SUPPLEMENTARY METHODS:

Experiment 1: Think/No-Think with unrelated bystander photographs

Notable deviations from the standard procedure set forth by experiment 1 and described in the Methods are listed in turn (see also Supplementary Fig. 1 and Supplementary Table 1 for a comparison of methods and participants).

Experiment 2: 24-hr delay

Special procedures. To further reduce the potential for task-related thoughts to perseverate into later trials, participants in experiment 2 made odd/even decisions based on the sum of two digits, a process carried out repeatedly in blocks of 7s—both before and after the bystander events. Catch trials were introduced following certain filler Think trials, with the constraint that they never abutted a critical bystander item. Blue asterisks indicated such events, prompting participants to vocalize the associate from the most recently displayed Think cue. Accuracy on these 19 catch trials was coded online by the experimenter.

Following the TNT phase and the administration of a short, generic exit questionnaire, participants were reminded to return the following day to complete a short, "unrelated" attention task. No constraints were placed on the participants' activities between sessions, save for asking them to again get at least 6hrs of sleep.

Experiment 3: Direct suppression vs. thought substitution

Materials. Thought substitutes for the 36 critical TNT responses were selected so as to be easily associable with their respective cue words but otherwise unrelated to the original response word (e.g., 'THERMOS' was the substitute for the response in the pairing 'PICNIC-HILL'). The substitutes were designed to avoid pre-existing semantic relationships with any other item in the

stimulus set, including the bystander imagery. Any given participant would go on to only learn 12 of those substitutes—the ones associated with No-Think cues in that person's counterbalancing condition. Six additional substitutes, used as fillers for all participants, were chosen in a similar manner.

Special procedures. After learning the original TNT parings to criterion, all participants—in both the direct suppression and thought substitution groups—then practiced the TNT task using fillers, first with a direct suppression strategy and then with a thought substitution strategy before being provided the list of substitute associates relevant to their counterbalancing condition (all participants were asked to refrain from thinking of the original associates during these presentations). We aimed to match the two groups perfectly on their training and strategy experiences prior to the critical TNT phase. Only then were they given divergent instructions.

Members of the direct suppression group were told that they would have to prevent both the original response and the substitute from coming to mind when presented with a No-Think cue. They were warned against generating any other diversionary thoughts. Members of the thought substitution group were instead asked to retrieve the associated substitute and focus on it the entire time the cue was presented. They were reminded to prevent the original associate from ever coming to mind and to push it out, if ever it did.

After the diagnostic questionnaire administered midway through the experiment, all participants were given a refresher on the full list of substitute pairings relevant to their counterbalancing condition. The thought substitution group was told to use this as an opportunity to reinforce their knowledge of the substitutes, whereas the direct suppression group was simply

told to passively view the words as they appeared. Both groups were warned against using this time to think about any of the original responses.

Experiment 4: Think/Think-Harder

Materials. Instead of suppressing a learned associate, participants in experiment 4 were cued to “Think Harder” about the associates. This multi-step task first required participants to retrieve the original associate, along with a learned alternate response. They were then to compare the strength of the semantic relationship between the original and learned alternate response to the relationship between the original response and a newly presented alternate. Should the latter relationship be judged the stronger of the two, they were to “switch” to the new alternate by updating their memory and using the new alternate as the comparison going forward (see elaborated *Procedure*, below). To this end, a new stimulus pool was created, consisting of 96 words selected to be unrelated to members of the standard set of TNT words or to the bystander photographs. From this pool, 12 words were randomly selected as “original alternates,” 52 as “repeated alternates,” and 32 as “critical-stay alternates.” For each of the original pairings, three further words were selected. These three were to be semantically related to the original TNT associate at a low, medium, and high degree. For example, ‘CHASE,’ ‘FLEAS,’ and ‘DOG’ became the three switch alternates for the response word ‘COLLIE’ in rank order. Informing these rankings were published norms[1] and subjective ratings from colleagues. The original, repeated, and critical-stay alternates remained fixed across participants, though the original and related alternates followed the standard counterbalancing procedure.

Special procedures. After learning the original Think/Think-Harder (T/TH) word pairs to criterion, participants were told that they would need to learn a number of alternate responses for some of the original ones. They were shown each of the critical Think-Harder pairings again, one

at a time, together with one of the pre-defined original alternates and a rating scale below it. The scale prompted participants to indicate the strength of the relationship between the alternate and the original response on a four-point scale ranging from ‘1’ (not at all related) to ‘4’ (highly related), having up to 10s to decide.

Once participants had completed this orienting task, they received the T/TH instructions and practiced implementing them before moving on to the critical T/TH phase. What follows is an example of what might be necessary on a Think-Harder trial. Participants might see the cue ‘LEAP’ in a red rectangle with ‘AGILE’ under it. In this case, they first had to recall the original associate of ‘LEAP’ (i.e., ‘BALLET’). Next, they had to decide whether ‘AGILE’ (presented at the bottom of the screen) was more related to ‘BALLET’ than was the original alternate (‘TIGHTS’), which they also had to recall. If they judged ‘AGILE-BALLET’ to be more related than ‘TIGHTS-BALLET,’ they were instructed to memorize the new alternate (‘AGILE’) and press the button labeled ‘yes’ to indicate that a switch had occurred. If not, they were to press ‘no’ and carry the original alternate (‘TIGHTS’) forward. Participants understood going into the T/TH phase that, at the end of the experiment, they would have to report the original trained associate, as well as the strongest alternate that they had come across during the intervening T/TH phase, should the cue come to be presented in a red rectangle during the intervening phase. Think trials took a form very similar to those in previous experiments. To match the physical responses, Think trials also required a button press; however, participants were instructed to always press the ‘no’ button to indicate that no switch was necessary.

The allowable duration of Think-Harder trials was increased to 4s, not inclusive of the standard pre- and post-cues. Think trials were expanded to match. Button responses were accepted only after the T/TH cue word was presented for at least 500ms. After a response, the

postcue was immediately presented and the experiment would advance. Think-Harder trials occurring right before or after critical bystanders were always accompanied by a unique critical-stay alternate, never a repeat or one of the alternates expected to prompt a switch response. Nor were they one of the 19 catch trials distributed throughout the T/TH phase to keep participants' attention focused. These special (catch) Think-Harder trials indicated to participants that their most recent button response was incorrect and that they should correct their behavior accordingly (i.e., maintain a previously rejected alternate or switch to a new one). The alternates for the catch trials were pre-selected to be of roughly equal relatedness to the Think-Harder associate, making the error feedback more plausible.

Experiment 5: Phonological rehearsal baseline

Materials. Pronounceable nonsense words were generated to replace the TNT cues in the phonological rehearsal condition using an automated password generator (http://www.adel.nursat.kz/apg/). Each nonsense word (e.g., 'TROAVI') replaced a single repetition of a TNT cue during the central phase. The final list of 432 critical nonsense words (3 syllables each, 5-11 letters in length) was divided into three sets, corresponding to Think, No-Think, and Baseline conditions (unique, filler nonsense words were generated in the same way). The sets were counterbalanced across participants. A corresponding set of 19 nonsense words was constructed by hand so that half would rhyme with their counterparts when read left to right and the other half would rhyme when read in the opposite direction. These linked sets would serve a role in the catch trials, described below.

Special procedures. Participants in both groups initially learned the standard set of TNT word pairs to criterion. However, participants in the phonological rehearsal condition would not go on to receive the standard TNT instructions. Instead, they practiced reading the nonsense

words either from left to right if they appeared in a green rectangle or from right to left if they appeared in a red rectangle, repeating as time permitted. Participants initially attempted this task aloud, only in later practice phases transitioning to subvocal rehearsal, as would be the practice throughout the main phase in which bystanders were introduced.

To keep participants honest and on task, catch trials would occasionally appear (though never before/after critical bystanders), prompting them to decide if a newly presented nonsense word (always read from left to right) rhymed with the nonsense word they had just been rehearsing (in the prescribed direction). They had up to 4s to make a yes/no response on the keyboard, after which the next trial appeared. Catch trials appeared 10 times after red trials and nine times after green trials, with 11 of the overall count intended to produce 'yes' responses. To match the influence of catch trials across the two groups, catch trials also appeared in matching serial positions for the group of participants that was given the standard TNT instructions with direct suppression. These catch trials always followed filler Think cues because they were uniquely compatible with the Think instructions, in that they prompted participants to say the associate for the previous retrieval cue into a microphone. Responses were coded for accuracy online.

Experiment 6: Word and picture recognition

Special procedures. Experiments 6a and 6b each comprised of data collected across two sites (St. Andrews and Oregon), using the same materials and nearly identical protocols. Data were collapsed across sites and a between-participants cohort factor was included in the statistical models. Unlike the St. Andrews cohort, which was specifically instructed to use a direct-suppression strategy, participants in the Oregon cohort were at liberty to choose their own suppression strategy so long as they never moved their eyes or attention away from the No-Think

cues, which they were to actively read (all participants, across studies, received instructions never to divert eyes/attention away from any of the cues, and their adherence to the instructions was monitored throughout). TNT pre- and postcues were not used in the Oregon cohort. Both groups received a two-step recognition test that assessed item and context memory for bystanders after the TNT phase.

Experiment 7: Neuroimaging

Special procedures. TNT trials lasted 4s, during which a red or green rectangle remained on the screen. Each trial consisted of a 750ms precue period, 3s during which the cue word was superimposed, and a 250ms postcue. The orienting task for bystander words was capped at 2500ms, with any remaining time following the button press filled with additional odd/even judgments, thereby preserving the absolute time lag between the onset of a bystander and the subsequent TNT trial. Odd/even (sum) buffer judgments occurred between each and every trial for a period of 2250ms-22s (including the 500ms warning cue) as determined by OptSeq2[2], which sought to optimize average efficiency for estimating the hemodynamic response to each of the aforementioned trial types.

Three optimized runs were selected, each of which allowed for two repetitions of each Think and No-Think cue and a single presentation of 13 unique bystander events. Runs were selected to ensure that there were always three bystanders in each of the 0-, 1-, and 2-suppression epoch conditions (because of the randomization procedure, two bystanders occasionally abutted—recall for these bystanders was ignored for the purposes of calculating the standard amnesic shadow effect). The status of each TNT cue (e.g., Think or No-Think) in the three orders was then counterbalanced, producing a final set of six orderings to be seen by every

participant in a randomized order. The six main TNT runs (each ~7.5 minutes in duration) were separated by rest periods of ~1 minute.

Participants viewed each of the learned TNT pairings for 1500ms as a refresher after entering the scanner. Then, to grant participants additional practice on the TNT task, functional scanning initialized with one warm-up run, which included a single repetition of all the critical Think and No-Think items, mixed with a number of filler bystanders. The range of durations for blocks of odd/even judgments was curtailed (from 2-6s); otherwise, the practice run simulated a critical TNT run they would later encounter. Although scanning took place during this short practice to acclimatize participants to the noise, the data were not suitable for analysis.

After the TNT phase, a high-resolution T2-weighted structural scan (~5m in duration) was obtained for each participant, during which, participants were simply asked to remain awake and still. The final context memory test for bystanders took place in the same room (outside the scanner) as was used for the initial TNT training. Items from the each of the three epoch conditions introduced in the six different TNT runs were distributed about equally across the test thirds in a block-randomized fashion, with the ordering of the thirds randomized for each participant. The first six test trials were drawn from filler items, which had been presented during the practice run. A short break was then allowed to answer any questions about the instructions, after which the rest of the test was administered without any breaks.

MRI acquisition and preprocessing. Imaging was conducted on a 3T Siemens TIM Trio MRI scanner at the MRC-CBU. An initial T1-weighted MPRAGE structural image (FOV = 256x240x192; 1mm isotropic voxels; TR = 2250ms; TE = 2.99ms; flip angle = 9°) was acquired before functional scanning commenced. For functional runs, a T2*-weighted echo-planar sequence was used (32 sequentially descending slices of 3mm thickness, slice gap = 25%, 64x64

matrix, 3x3mm in-plane resolution, slices were collected in an oblique orientation ~30° from the AC-PC line, upwards anteriorly, with TR = 2s, TE = 30ms, and flip angle = 78°). A total of 238 functional volumes were collected for each TNT run, although the first 10—all recorded prior to commencing the behavioral paradigm—were excluded from analysis to stabilize T1 magnetization.

Raw DICOM files were converted to FSL NIfTI format using dcm2nii software (http://www.cabiatl.com/mricro/mricron/dcm2nii.html). Preprocessing and statistical analyses of the functional data were conducted using FSL version 4.1.2[3]. The six scanning runs were modeled separately at the first level using FSL's FEAT (version 5.98) and were subjected to the following standard pre-processing steps: motion correction using MCFLIRT[4], slice-timing correction using Fourier-space time-series phase-shifting, non-brain removal using BET[5], spatial smoothing using an 8mm FWHM Gaussian kernel; grand-mean intensity normalization of the dataset by a single multiplicative factor; and 1/120Hz high-pass temporal filtering (Gaussian-weighted least-squares straight line fitting, with sigma = 60s). FLIRT was used to carry out registration to high-resolution structural and standard space images [T1-weighted, Montreal Neurological Institute's template brain with 2mm resolution (MNI152)][4,6], with further refinements made by FNIRT nonlinear registration[7,8].

fMRI analysis. FMRI data processing was conducted using FSL's FEAT (version 5.98). Think, No-Think, correctly categorized bystanders segregated by their surrounding context, and any bystanders incorrectly categorized by the participant were binned separately and modeled as delta functions at cue-onsets before being convolved with a canonical hemodynamic response function (specifically, a gamma kernel with phase shift = 0, M_{lag} = 6s; SD_{lag} = 3). Separate regressors for motion parameters were included. Temporal filtering was applied to the model to

match the data. Time-series statistical analyses were carried out using a generalized linear model (FILM) with local autocorrelation correction/pre-whitening[9]. For each participant, a higher-level analysis across the six TNT runs was carried out using a fixed-effects model, by forcing the random effects to zero in FLAME[10,11]. Group analyses were conducted using FLAME (stage 1) analysis of mixed effects[10,11]. Whole-brain statistical maps for both the Think > No-Think and No-Think > Think contrasts were created with a cluster-forming threshold of $z > 2.3$ and a cluster significance threshold of $P < 0.05$, according to Gaussian random field theory[12].

Anatomical regions of interest (ROIs) were defined according to the Harvard-Oxford probabilistic atlas in standard space, thresholded at 40% probability. Analyses largely focused on the *a priori* anatomical (bilateral) hippocampal ROI, which contained 1327 voxels. For completeness, an exploratory analysis also examined separate right- (691 voxels) and left-lateralized (636 voxels) hippocampal masks. Aspects of the anatomically defined left hippocampus that also appeared in the whole-brain Think > No-Think main effect contrast were examined in a similarly exploratory fashion as a separate ROI (this area of overlap comprised 106 voxels). FSL's Featquery tool[3] was used to extract from these ROIs the mean contrasts of parameter estimates (COPEs) comparing Think and No-Think tasks. COPE values extracted from the hippocampal ROIs were entered into one-sample, *t*-tests to examine the *a priori* prediction that No-Think activity was reduced relative to Think trial activity within the hippocampal ROIs, as previously observed[13-19]. While our *a priori* prediction was clearly directional, we report the findings from two-tailed tests for consistency.

The COPE values extracted from hippocampal and prefrontal regions modulated by the TNT task were also entered into robust brain-behavior correlation analyses, across participants. The calculation of the primary behavioral correlate (the recognition shadow effect, defined here

as the behavioral difference in context memory accuracy across the 0 epoch and 2 epoch conditions) was restricted to bystanders that were both correctly categorized (with associated reaction times >400ms to account for guessing/mistaken button responses) during the animacy/pleasantness orientating task and surrounded by a mean odd/even buffer lag (across the pre- and post-bystander windows) less than or equal to the mean buffer lag across the six behavioral experiments (5.2s). The Pearson skipped-correlation method[20] adopted for present purposes down-weights/removes outliers that could otherwise exert disproportionate influence on the measured relationship, especially when the sample size is limited. Bivariate outliers were identified analytically for each analysis reported by the Robust Correlation Toolbox for Matlab, without manual intervention, and were similarly stricken from the associated scatterplots. In accordance with the toolbox's design, we determined when to reject the null hypothesis of independence based on the percentile bootstrap confidence interval (i.e., if the 95% c.i. includes 0, the null hypothesis cannot be rejected), a method which is more robust against heteroscedasticity than the traditional *t*-test[20].

SUPPLEMENTARY REFERENCES:

1 Polyn, S. M., Norman, K. A. & Kahana, M. J. A context maintenance and retrieval model of organizational processes in free recall. *Psychol. Rev.* **116**, 129-156 (2009).

2 Dale, A. M. Optimal experimental design for event-related fMRI. *Hum. Brain Mapp.* **8**, 109-114 (1999).

3 Smith, S. M. *et al.* Advances in functional and structural MR image analysis and implementation as FSL. *NeuroImage* **23, Supplement 1**, S208-S219 (2004).

4 Jenkinson, M., Bannister, P., Brady, M. & Smith, S. M. Improved optimisation for the robust and accurate linear registration and motion correction of brain images. *NeuroImage* **17**, 825-841 (2002).

5 Smith, S. M. Fast robust automated brain extraction. *Hum. Brain Mapp.* **17**, 143-155 (2002).

6 Jenkinson, M. & Smith, S. M. A global optimisation method for robust affine registration of brain images. *Med. Image Anal.* **5**, 143-156 (2001).

7 Andersson, J. L. R., Jenkinson, M. & Smith, S. M. Non-linear registration aka spatial normalisation, technical report. Report No. TR07JA2, (University of Oxford, Nuffield Department of Clinical Neurosciences, Centre for Functional MRI of the Brain, Oxford, England, 2007).

8 Andersson, J. L. R., Jenkinson, M. & Smith, S. M. Non-linear optimization, technical report. Report No. TR07JA1, (University of Oxford, Nuffield Department of Clinical Neurosciences, Centre for Functional MRI of the Brain, Oxford, England, 2007).

9 Woolrich, M. W., Ripley, B. D., Brady, M. & Smith, S. M. Temporal autocorrelation in univariate linear modeling of FMRI data. *NeuroImage* **14**, 1370-1386 (2001).

10 Beckmann, C. F., Jenkinson, M. & Smith, S. M. General multilevel linear modeling for group analysis in FMRI. *NeuroImage* **20**, 1052-1063 (2003).

11 Woolrich, M. W., Behrens, T. E. J., Beckmann, C. F., Jenkinson, M. & Smith, S. M. Multilevel linear modelling for fMRI group analysis using Bayesian inference. *NeuroImage* **21**, 1732-1747 (2004).

12 Worsley, K. J., Evans, A. C., Marrett, S. & Neelin, P. A three-dimensional analysis for CBF activation studies in human brain. *J. Cereb. Blood Flow Metab.* **12**, 900-918 (1992).

13 Anderson, M. C. *et al.* Neural systems underlying the suppression of unwanted memories. *Science* **303**, 232-235 (2004).

14 Depue, B. E., Curran, T. & Banich, M. T. Prefrontal regions orchestrate suppression of emotional memories via a two-phase process. *Science* **317**, 215-219 (2007).

15 Levy, B. J. & Anderson, M. C. Purging of memories from conscious awareness tracked in the human brain. *J. Neurosci.* **32**, 16785-16794 (2012).

16 Benoit, R. G. & Anderson, M. C. Opposing mechanisms support the voluntary forgetting of unwanted memories. *Neuron* **76**, 450-460 (2012).

17 Benoit, R. G., Hulbert, J. C., Huddleston, E. & Anderson, M. C. Adaptive top-down suppression of hippocampal activity and the purging of intrusive memories from consciousness. *J. Cognit. Neurosci.* **27**, 96-111 (2015).

18 Gagnepain, P., Henson, R. N. & Anderson, M. C. Suppressing unwanted memories reduces their unconscious influence via targeted cortical inhibition. *Proc. Natl. Acad. Sci. USA* **111**, E1310-1319 (2014).

19 Anderson, M. C. & Hanslmayr, S. Neural mechanisms of motivated forgetting. *Trends Cogn. Sci.* **18**, 279-292 (2014).

20 Pernet, C. R., Wilcox, R. R. & Rousselet, G. A. Robust correlation analyses: false positive and power validation using a new open source Matlab toolbox. *Front. Psychol.* **3** (2013).
